# Photoacoustic Techniques for Trace Gas Sensing Based on Semiconductor Laser Sources

**DOI:** 10.3390/s91209616

**Published:** 2009-12-02

**Authors:** Angela Elia, Pietro Mario Lugarà, Cinzia Di Franco, Vincenzo Spagnolo

**Affiliations:** Laboratorio Regionale CNR-INFM “LIT ^3^”, Dipartimento Interateneo di Fisica “M. Merlin”, Università and Politecnico of Bari, Bari, I-70126, Italy; E-Mails: lugara@fisica.uniba.it (P.M.L.); cinzia.difranco@fisica.uniba.it (C.D.F.); spagnolo@fisica.uniba.it (V.S.)

**Keywords:** gas sensing, photoacoustic spectroscopy, quartz enhanced photoacoustic spectroscopy

## Abstract

The paper provides an overview on the use of photoacoustic sensors based on semiconductor laser sources for the detection of trace gases. We review the results obtained using standard, differential and quartz enhanced photoacoustic techniques.

## 1. Introduction

Photoacoustic spectroscopy (PAS) is a widely used method for trace gas detection. It is based on the photoacoustic (PA) effect, [1], *i.e.*, the conversion of light to sound in all materials (solids, liquids and gases). Viengerov [2] used the PA technique for the first spectroscopic gas analysis in 1938; he studied blackbody infrared light absorption in gases to detect the concentrations in a mixture. Later, Luft [3] enhanced the detection sensitivity of the PA system to parts per million (ppm) levels. This was possible even with radiation sources of low spectral brightness since PAS directly measures the absorbed energy in a sample, rather than the transmitted radiation as in the conventional spectroscopies. The increased interest in PAS was due to the development of new high power laser sources and electronics, *i.e.*, sensitive microphones and lock-in detection and amplification schemes. Kerr and Atwood [4] used for the first time, a continuous wave (cw) CO_2_ laser, as radiation source, to detect carbon dioxide, buffered in nitrogen. Subsequently, Kreuzer [5] reported the detection of methane with a limit of 10 parts per billions (ppb), using a HeNe laser, and initiated many activities in this field. PA systems devoted to gas samples achieve now sub-ppb detection limits and have linear response in a wide range of gas concentrations, covering 6–8 orders of magnitude.

PAS generation and detection in gases was first studied by Kreuzer [6]. In general, two main steps must be considered. The first step concerns the absorption of modulated light of appropriate wavelength by molecules and heat release in the gas sample due to non-radiative relaxation (molecular collisions) of the excited states. The non-radiative relaxation process in optically excited gaseous samples occurs when the relaxation time can compete with the radiative lifetime of the excited energy levels. Radiative decay has a characteristic 10^−7^ s lifetime at visible wavelengths, as compared with 10^−2^ s at 10 μm. For non-radiative decay the lifetime values depend on the pressure (decay time is inversely proportional to the pressure) and can vary strongly at atmospheric pressures (10^−3^–10^−8^ s). The second step consists of modulating the radiation source at an acoustic frequency, and therefore the temperature changes periodically giving rise to a pressure wave. The photoacoustic signal is traditionally detected via a resonant acoustic cell equipped with a sensitive microphone [7]. Recently alternative transducers, *i.e.*, quartz tuning forks [8], optimised capacitive microelectromechanical systems microphones [9] or a silicon cantilever [10], have been demonstrated. In this paper we will provide a brief overview of semiconductor laser-based photoacoustic sensors for the detection of trace gases; in particular, the results obtained using standard, differential and quartz enhanced photoacoustic techniques will be reported.

## 2. Semiconductor Lasers

The radiation sources for sensitive and selective PAS applications must exhibit the following characteristics: (i) high optical power; (ii) narrow linewidth; (iii) single mode operation; (iv) low source noise and low amplitude fluctuations; (v) high stability and reliability; (vi) compact and robust overall sensor package.

The whole spectral range from the visible to the infrared can be covered by semiconductor lasers. Commercially available gallium arsenide and indium phosphide lasers operate in the 0.5–2 μm spectral range and overlap the spectra of vibrational overtones, which show two or three order of magnitude weaker cross-section than the fundamental vibrations [11].

In the mid infrared (MIR) region source options include gas lasers (CO, CO_2_), lead-salt diode lasers, coherent sources based on difference frequency generation (DFG), optical parametric oscillators (OPOs), quantum and interband cascade lasers. Among them, quantum cascade (QC) and interband cascade (IC) lasers represent an effective choice for PA spectroscopy with ultra-high resolution and sensitivity [12]. The highest performance distributed feedback (DFB) QC lasers are based on type-I intersubband transitions in InGaAs/InAlAs heterostructures [13-16]. Typical emission wavelengths can be varied in the range 3.5–24 μm with record optical power up to few Watts [17].

QCLs are characterized by a limited tuning range: approximately 3–4 cm^−1^ by adjusting the injection current or up to approximately 20 cm^−1^ by controlling the temperature of the laser chip [18]. This limitation has been overcome combining quantum-cascade lasers with external-cavity geometries (External cavity QCL) resulting in MIR quantum-cascade lasers with narrowband output, high power and a broad tuning range exceeding 100 cm^−1^ [19].

More recently, interband cascade lasers based on type-II interband transition have been reported in the 3–5 μm region [20]. These sources give the possibility to access the spectral region of important absorption features (C-H and O-H stretch vibrations) near 3,000 cm^-1^, overcoming another limitation of type-I QCLs.

Semiconductor lasers can be easily and rapidly modulated, through a modulation of their injection current. Laser modulation capability is a key issue for high sensitivity gas detection and many of the most performant techniques for trace gas monitoring take advantage of laser modulation for noise reduction and high sensitivity detection.

Laser intensity, wavelength and/or frequency modulation techniques exploit the fact that the frequency-modulation of a laser, passing through a frequency-dependent absorption medium, results in a transmitted power variation at the modulation frequency and its harmonics. Phase-sensitive detection at frequencies beyond the range of technical noise sources is thus possible.

Wavelength modulation (WM) scheme conventionally describes the case where the modulation frequency is much less than the width of the spectral feature of interest and the modulation index is high. Generally, this corresponds to modulation frequencies from a few kilohertz to a few megahertz. In the case of frequency modulation (FM) technique the modulation frequency is comparable to, or greater than, the spectral width of the target feature and the modulation index is sufficiently low that only the first two sidebands of the modulated laser spectrum have significant amplitude. In this case, modulation frequencies are typically in excess of 100 MHz. To access fundamental molecular-vibrational bands in the MIR FM schemes have been demonstrated with quantum cascade lasers [21], which offer similar advantages to diode lasers in terms of ease of modulation. MIR WM with milliwatt-level powers has also been demonstrated by difference frequency generation between amplified near-IR diode lasers, whose modulation then transfers to the MIR output [22].

As the PA signal is directly proportional to the laser power, PAS has been primarily implemented using high power mid-infrared gas lasers (mainly CO_2_ lasers), in order to achieve detection limits in the ppb range. In that case, intensity modulation (IM) technique has been used, either via an external modulation (mechanical chopper), or with direct modulation of a radio frequency-excited CO_2_ laser [7]. Similarly to gas lasers, semiconductor lasers may be intensity-modulated. This may be accomplished either using a mechanical chopper or, more generally, by directly switching on and off the laser injection current. These lasers also offer the possibility to be wavelength modulated, by modulating their injection current with a reduced amplitude in order to tune the laser on-line and off-line. Such a modulation scheme may give some advantages. For example, window noise and wall noise are efficiently suppressed by WM and harmonic detection [23]. WM may also be slightly more efficient than IM in the generation of the PA signal [24].

## 3. Standard PAS

The standard approach to detecting the acoustic signal generated by the modulated laser radiation in a weakly absorbing gas utilizes an acoustic resonator filled with the gas [25-27].

The PA signal measured by the microphone, is given by:

(1) S=C⋅P(λ)⋅α(λ)

where C is the cell constant in the unit of Vcm/W, P the optical power of the laser source and *α* the absorption coefficient which is related to the gas concentration (*N*, number density of molecules) and absorption cross section (σ) by *α* = *Nσ*. The cell constant depends on the geometry of the sample cell, the beam profile, the microphone response and the nature of the acoustic mode.

**Figure 1.**
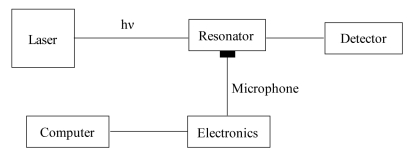
Typical PA spectrometer.

A conventional photoacoustic spectrometer consists of a radiation source, a modulator, a photoacoustic cell and a signal acquisition and processing equipment. A schematic diagram is shown in (Figure 1).

The modulated laser radiation passes through the PA cell filled with the gaseous sample and enters a power monitor. The absorption of modulated light generates an acoustic signal in the cell, used for photoacoustic measurements. The PA signal can be amplified by tuning the modulation frequency to one of the acoustic resonances of the sample cell. In this resonant case the cell works as an acoustic amplifier; the absorbed laser power is accumulated in the acoustic mode of the resonator for *Q* oscillation periods, where *Q* is the quality factor, typically in the range of 10–300. The excited sound waves can be detected by an electret or condenser microphone and the microphone signal is measured by a lock-in amplifier.

Continuous-wave single-frequency diode lasers in the near IR have been successfully applied in PA spectroscopy. These lasers overlap with the spectra of vibrational overtones which have small absorption cross sections. Resonant PA cells or optical fiber amplifier have been developed [28-31] to enhance the sensitivity. A selection of the results reported so far is summarized in (Table 1).

The implementation of high power DFB-QCL excitation in the MIR fundamental absorption region (fingerprint region) leads to considerably improved trace gas detection sensitivity. Since 1999, when DFB-QCL were demonstrated for the first time in trace gas sensing applications [32], a large number of gas species have been detected as reported in (Table 2) [33-39].

## 4. Differential PAS

**Figure 2.**
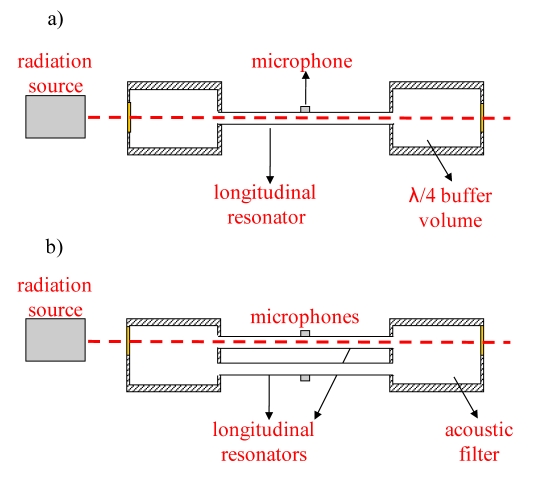
(a) Resonant PA cell with one resonator (typical resonator length of 100–300 mm) and two buffer volumes (half resonator length). (b) Differential PA cell. with two resonator tubes and acoustic filters

PAS is ideally a background-free technique, since the signal is generated only by the absorbing gas. However, background signals can originate from nonselective absorption of the gas cell windows (coherent noise) and external acoustic (incoherent) noise. In order to obtain an efficient noise reduction and, thus, to improve the signal to noise ratio (SNR) performances, different designs for PA cell have been proposed [40,41], *i.e.*, resonant cell with acoustic buffers (Figure 2a), “windowless” and differential cell (Figure 2b).

Differential PAS represents an effective choice; many differential photoacoustic schemes have been reported in literature. A differential cell (Figure 2b), specially designed to suppress flow and windows noise, has been introduced by Miklós *et al.* [41]. In order to reduce the flow noise and external electromagnetic disturbances, the PA cell is characterized by a symmetrical design and consists of two acoustic resonators (5.5 mm in diameter, 40 mm in length) placed between two band-stop acoustic filters. Each tube is equipped with a microphone with the same responsivity around the resonance frequency of the cell (f < 4 kHz). Since the laser light excites only one of the two resonator, the difference of the two microphones provides the actual measurement removing any noise components that are coherent in both resonator tubes. A large number of gas species (NH3, CH_4_, CH_2_O, N_2_O, NO_2_, O_3_) have been detected using this differential PA cell in combination with different radiation sources (near-infrared diode lasers, optical parametric oscillators, QCLs,) with detection limits in the range 1–200 ppb [37,42-46,36,39]. A selection of the results with quantum cascade lasers is reported in (Table 2 [36,37,39]). Recently, a novel photoacoustic scheme, the differential mode excitation photoacoustic spectroscopy, has been reported in conjunction with blackbody light sources [47] and near infrared light emitting diode [48].

## 5. QEPAS

Quartz enhanced photoacoustic spectroscopy (QEPAS) is an alternative way to detect weak PA excitation [49,50]. The basic idea of QEPAS is to invert the common PAS approach and accumulate the acoustic energy not in a gas filled cell but in a sharply resonant acoustic transducer. Such an approach removes restrictions imposed on the gas cell by acoustic resonance conditions. The transducer can be positioned in the acoustic near-field zone of the excitation optical beam; in this case the gas enclosure is optional and helps only to separate the gas sample from the environment and to control its pressure. A well-suited material for a resonant high-*Q* microphone is a piezoelectric quartz crystal. Usually it is a quartz tuning fork (QTF) with a resonant frequency close to 32,768 (*i.e.*, 2^15^) Hz. The mode at this frequency corresponds to a symmetric vibration (the prongs move in opposite directions). A mechanical deformation of the QTF prongs induces electrical charges on its electrodes and a practical way to acquire the QTF electrical response is to utilize a trans-impedance amplifier. These QTFs are widely used for atomic-force and optical near-field microscopy. A typical watch QTF has a value of Q = 20,000 or higher in vacuum and of Q = 8,000 at normal atmospheric pressure. Therefore the corresponding energy accumulation time at atmospheric pressure is of the order of few hundreds of ms, which is a noticeably longer time than any practical gas-filled resonator can provide. The QEPAS response (S) to optical absorption is described by the following equation:

$$S=k\frac{\alpha lCPQ}{fV}$$

where *α* is the absorption coefficient per unit concentration of the target trace gas species, *l* is the resonator length, *C* is the concentration of the target species, *P* is the applied optical power, *f* is the QEPAS sound frequency, *V* is the resonator volume and *k* is a constant describing other system parameters.

An important feature of the QEPAS is its immunity to background acoustic noise. This is due to the following reasons: (i) the ambient noise shows a 1/*f* dependence and is very low above 10 kHz; (ii) the acoustic wavelength in air is about 1 cm at 32 kHz and is longer at lower frequencies and acoustically a quartz TF is a quadrupole. Therefore, any external sound waves produce a force in the same direction upon the two TF prongs positioned at an ∼1 mm distance. This does not excite the piezoelectrically active mode in which the two prongs move in opposite directions; (iii) the width of the TF resonance at normal pressure is ∼4 Hz, and only frequency components in this narrow spectral band can produce efficient excitation of the TF vibration.

The QEPAS results are also sensitive to the gas sample pressure since: (i) the Q-factor decreases at higher pressures, (ii) the peak optical absorption varies with pressure, (iii) the V-T energy transfer relaxation rate from vibrationally to translational degrees of freedom is faster at higher pressures, resulting in more efficient sound excitation and the acoustic resonator enhancement factor changes with pressure [51-54]. Thus, great care should be used to optimize the laser wavelength modulation amplitude Δλ as a function of the pressure for the highest signal; an optimum value has been demonstrated to be Δλ ≈ 2 × FWHM of the target absorption line.

The majority of reported QEPAS-based sensor configurations include a spectrophone (the module for detecting laser-induced sound) consisting of a QTF and a microresonator composed of a pair of thin tubes, the aim of this two tubes is to increase the effective interaction length between the radiation-generated sound and the TF [55]. The tubes, made of silica, are typically 2.45 mm long with a 0.32 mm inner diameter, aligned perpendicular to the TF plane. The distance between the free ends of the tubes is equal to half wavelength of sound in air at 32.75 kHz, thus satisfying the resonant condition [26]. Experiments have shown that the microresonator yields a signal gain of 10 to 20 [50,55,56].

**Figure 3.**
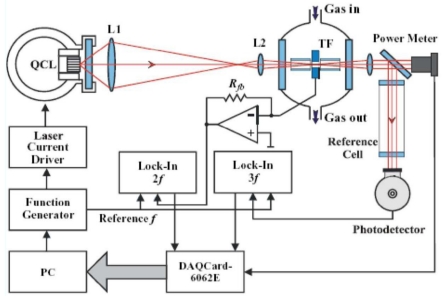
Schematic of the QEPAS trace gas sensor using a quantum cascade laser as an excitation source. (Reproduced with kind permission form Springer Science and Business Media [52]).

QEPAS studies reported to date have been performed mostly using a wavelength modulation technique at half the QTF resonance frequency with second-harmonic (2f) detection especially for target analytes with well-resolved absorption lines. IM approaches at the QTF frequency and detection at the same frequency has been employed in QEPAS systems [57-60] typically for molecular species with unresolved congested spectra. A comparison between these two approaches is reported in references [55] and [61]. A schematic of a QEPAS-based gas sensor with WM configuration is shown in Figure 3. The sensor consists of the three subsystems: a control and data processing electronics unit, a transimpedance amplifier with an electronic switch and a set of optical components including the excitation laser, a reference cell with a photodetector and a QTF. A continuous wave laser was used as a photoacoustic excitation source. The laser beam is focused between the prongs of the QTF and its wavelength is modulated at *f*_m_ = *f*_0_*/*2 frequency, where *f*_0_ is the QTF resonant frequency. The laser wavelength is modulated by adding an AC component to the device voltage. The dc component of the laser driver input *V* determines the laser central wavelength. The 3*f* component of the photodetector signal is measured and the voltage is adjusted proportionally to maintain the laser wavelength at the centre of the absorption line while a lock-in amplifier is used to demodulate the QTF response at *f*_0_. Spectral data can be acquired by scanning the wavelength. To increase the effective interaction length between the radiation-induced sound and the QTF, an acoustic gas-filled resonator can be added similarly to the traditional PAS approach.

A large number of gas species have been detected using QEPAS approach and the results reported so far are summarized in (Table 3) [61]. The normalized noise equivalent absorption coefficient measured to date using QEPAS are comparable to the best conventional PA results and experimental study of the long-term stability of a QEPAS sensors showed that the sensors exhibit low drift, which allows long term data averaging thus allowing a significant improvement of the signal-to-noise ratio in concentration measurements.

## 4. Conclusions

In this paper we have reported the state of art laser-based photoacoustic sensors for trace gas detection. Infrared tunable semiconductor lasers represent the ideal radiation sources for gas sensing thanks to their excellent spectroscopic and technical properties, *i.e.*, narrow linewidth, tunability, reliability and room-temperature operation. They include near-infrared gallium arsenide and indium phosphide laser diodes and quantum cascade lasers in the mid-infrared.

Compact, sensitive and selective PA sensors based on the semiconductor lasers have been demonstrated to be effective in a great number of applications, *i.e.* environmental monitoring (e.g., NO_x_, CH_4_, CO_2_, H_2_CO), industrial emission measurements, chemical analysis and control for manufacturing processes (e.g., NH_3_, HMDS, C_2_H_2_, H_2_CO, C_2_H_5_OH), detection of medically important molecules (e.g., NO, CO_2_, NH_3_), toxic gases and explosives.

The detection limits and performances obtained with standard and differential photoacoustic schemes have been summarized. The results reported with recently developed approach based on a quartz tuning fork as a sharply resonant acoustic transducer have been discussed, as well.

## Figures and Tables

**Table 1.** PA detection of trace gases with NIR diode lasers.

**Molecule [Ref.]**	**Laser source**	**Integration time**	**Detection limit** **(SNR = 1)**	**PA cell**
CO_2_ [28]	Wavelength modulated DFB diode laser; λ = 1.573 μm, P = 4.5 mW	100 s	30 ppm	Dual-microphone resonant cell f = 1.456 KHz
C_2_H_2_ [29]	Wavelength modulated DFB diode laser; λ= 1.51 μm, P = 3.5 mW	3 ms	10 ppm	Resonant on first longitudinal mode f = 2.1 KHz
CH_4_ H_2_O HCl [30]	Fibre-pigtailed laser diodes, λ = 1.651 μm (P = 18 mW); λ = 1.3686 μm (P = 22 mW); λ = 1.7379 μm (P = 16 mW)	10 s	CH_4_: 27 ppb H_2_O: 5 ppb HCl: 10 ppb	Multi-gas PA resonant cell on first longitudinal mode f = 1 KHz
NH_3_ [31]	Fibre amplified diode laser; λ = 1.53 μm; P = 500 mW	10 s	6 ppb	Resonant on first longitudinal mode f = 900 Hz

**Table 2.** PA detection of trace gases with QC lasers.

**Molecule [Ref.]**	**Laser source**	**Integration time**	**Detection limit** **(SNR = 1)**	**PA cell**
NO [33]	RT Pulsed DFB QCL; λ = 5.3 μm, P = 2 mW	10 s	150 ppb	4-microphones resonant cell on first longitudinal mode f = 1.38 KHz
CH_2_O [34]	RT Pulsed DFB QCL; λ = 5.6 μm, P = 4 mW	10 s	150 ppb	4-microphones resonant cell on first longitudinal mode f = 1.38 KHz
NO_2_ [35]	RT cw QCL (external grating cavity) λ = 6.25 μm, P = 300 mW	1 s	0.5 ppb	Resonant on first longitudinal mode f = 1.8 KHz
NO_2_ N_2_O [36]	RT Pulsed DFB QCLs; λ = 6.2 μm (P = 5 mW); λ = 8 μm (P ∼ 10 mW)	-	∼80 ppb	Differential PA cell resonant on first longitudinal mode f = 3.8 KHz
NH_3_ [37]	RT Pulsed DFB QCL; λ = 9.6 μm, P = 2 mW	-	30 ppb	Differential PA cell resonant on first longitudinal mode f = 3.8 KHz
HMDS* [38]	Cryogenically cooled pulsed Fabry-perot QCL λ= 8.4 μm	10 s	200 ppb	4-microphones resonant cell on first longitudinal mode f = 1.38 KHz
O_3_ [39]	RT Pulsed DFB QCL; λ = 9.5 μm, P ≈ mWs	-	100 ppb	Differential PA cell resonant on first longitudinal mode f = 3.8 KHz

* HMDS: hexamethyldisilazane

**Table 3.** QEPAS detection of trace gases.

**Gas species**	**Frequency** **[cm^−1^]**	**Pressure** **[Torr]**	NNEA* [cm^−1^W/Hz^1/2^]	**Power** **[mW]**	NEC**(τ = 1s) **[ppm]**
H_2_O (N_2_)	7,306.75	60	1.9 × 10^−9^	9.5	0.09
HCN(air: 50% RH)	6,539.11	60	< 4.3 × 10^−9^	50	0.16
C_2_H_2_ (N_2_)	6,523.88	720	4.1 × 10^−9^	57	0.03
NH_3_ (N_2_)	6,528.76	575	3.1 × 10^−9^	60	0.06
C_2_H_4_ (N_2_)	6,177.07	715	5.4 × 10^−9^	15	1.7
CH_4_ (N_2_+1.2% H_2_O)	6,057.09	760	3.7 × 10^−9^	16	0.24
CO_2_ (breath)	6,361.25	150	8.2 × 10^−9^	45	40
H_2_S	6,357.63	780	5.6 × 10^−9^	45	5
CO_2_ (N_2_+1.5% H_2_O)	4,991.26	50	1.4 × 10^−8^	4.4	18
CH_2_O	2,804.90	75	8.7 × 10^−9^	7.2	0.12
CO (N_2_)	2,196.66	50	5.3 × 10^−7^	13	0.5
CO (propylene)	2,196.66	50	7.4 × 10^−8^	6.5	0.14
N_2_O (air+5% SF6)	2,195.63	50	1.5 × 10^−8^	19	0.007
C_2_H_5_OH (N_2_)	1,934.2	770	2.2 × 10^−7^	10	90
C_2_HF_5_ (N_2_)	1,208.62	770	7.8 × 10^−9^	6.6	0.009
NH_3_ (N_2_)	1,046.39	110	1.6 × 10^−8^	20	0.006

* NNEA – normalized noise equivalent absorption coefficient.

** NEC – noise equivalent concentration for available laser power and τ = 1 s time constant, 18 dB/oct filter slope.
